# *Ganoderma* spore lipid ameliorates docetaxel, cisplatin, and 5-fluorouracil chemotherapy-induced damage to bone marrow mesenchymal stem cells and hematopoiesis

**DOI:** 10.1186/s12906-024-04445-x

**Published:** 2024-04-12

**Authors:** Haohui Lin, Manhon Chung, Jingchun Sun, Yi Yang, Li Zhang, Xiaohua Pan, Minghui Wei, Sa Cai, Yu Pan

**Affiliations:** 1 Health Science Center, The 2nd Affiliated Hospital of Shenzhen University, Shenzhen University, Shenzhen, China; 2 Department of Plastic and Reconstructive Surgery, Shanghai Ninth People’s Hospital, Shanghai Jiao Tong University School of Medicine, Shanghai, China; 3 Department of Head and Neck Surgical Oncology, National Cancer Center, National Clinical Research Center for Cancer, Cancer Hospital & Shenzhen Hospital, Chinese Academy of Medical Sciences and Peking Union Medical College, Shenzhen, China

**Keywords:** Head and neck squamous cell carcinoma, Chemotherapy, Docetaxel, cisplatin, and 5-fluorouracil, *Ganoderma* spore lipid, Bone marrow mesenchymal stem cell, Hematopoiesis

## Abstract

**Background:**

A triplet chemotherapy regimen of docetaxel, cisplatin, and 5-fluorouracil (TPF) is used to treat head and neck squamous cell carcinoma; however, it is toxic to bone marrow mesenchymal stem cells (BMSCs). We previously demonstrated that *Ganoderma* spore lipid (GSL) protect BMSCs against cyclophosphamide toxicity. In this study, we investigated the protective effects of GSL against TPF-induced BMSCs and hematopoietic damage.

**Methods:**

BMSCs and C57BL/6 mice were divided into control, TPF, co-treatment (simultaneously treated with GSL and TPF for 2 days), and pre-treatment (treated with GSL for 7 days before 2 days of TPF treatment) groups. In vitro, morphology, phenotype, proliferation, senescence, apoptosis, reactive oxygen species (ROS), and differentiation of BMSCs were evaluated. In vivo, peripheral platelets (PLTs) and white blood cells (WBCs) from mouse venous blood were quantified. Bone marrow cells were isolated for hematopoietic colony-forming examination.

**Results:**

In vitro, GSL significantly alleviated TPF-induced damage to BMSCs compared with the TPF group, recovering their morphology, phenotype, proliferation, and differentiation capacity (*p* < 0.05). Annexin V/PI and senescence-associated β-galactosidase staining showed that GSL inhibited apoptosis and delayed senescence in TPF-treated BMSCs (*p* < 0.05). GSL downregulated the expression of caspase-3 and reduced ROS formation (*p* < 0.05). In vivo, GSL restored the number of peripheral PLTs and WBCs and protected the colony-forming capacity of bone marrow cells (*p* < 0.05).

**Conclusions:**

GSL efficiently protected BMSCs from damage caused by TPF and recovered hematopoiesis.

**Supplementary Information:**

The online version contains supplementary material available at 10.1186/s12906-024-04445-x.

## Background

Chemotherapy is a crucial treatment for head and neck squamous cell carcinoma (HNSCC) [1]. The preferred chemotherapy regimen for HNSCC is triplet docetaxel, cisplatin, and 5-fluorouracil (TPF), which improves the progression-free and overall survival of patients [2, 3]. However, TPF exhibits extreme hematopoietic toxicity and can damage the hematopoietic microenvironment (HME), leading to myelosuppression in patients with HNSCC [4–6]. The HME, also known as the niche, comprises mesenchymal stem cells (MSCs), stromal cells, and a specific extracellular matrix [7]. Hematopoietic stem cells (HSCs) are regulated by various hematopoietic signals from the HME that help maintain the balance between self-renewal and differentiation [7]. Therefore, disruption to the HME leads to hematopoietic disorders. Several studies have reported that HME homeostasis largely relies on bone marrow mesenchymal stem cells (BMSCs) [8, 9]. BMSCs participate in the construction of HME and directly regulate hematopoiesis by secreting hematopoietic stimulating factors and providing cellular contact [8, 9]. In contrast, damage to BMSCs interferes with the viability and function of HSCs and their depletion may even cause irreversible myelosuppression that severely impairs hematopoiesis [10, 11]. However, many chemotherapeutic drugs are cytotoxic to BMSCs, including docetaxel, cisplatin, and 5-fluorouracil [12–15]. 5-Fluorouracil damages BMSCs by inducing oxidative stress, resulting in decreased levels of hematopoietic stimulating factors, including stem cell factor, stromal cell-derived factor, and granulocyte-macrophage colony-stimulating factor [15]. Taxane compounds, such as docetaxel and paclitaxel, trigger premature senescence in BMSCs [13]. Moreover, cisplatin promotes BMSC apoptosis by activating the PI3K-AKT-mTOR pathway and increasing the expression of caspase-3 and p21 [14]. Therefore, TPF can damage BMSCs, which may accelerate the HME and impede hematopoiesis. Hence, protecting BMSCs may help ameliorate hematopoietic toxicity and myelosuppression induced by TPF.

Traditional Chinese agents have positive effects on the hematopoietic system. Some herbal extracts or compounds can relieve the hematopoietic toxicity of chemotherapeutic drugs by protecting BMSCs [14–17]. *Ganoderma lucidum* is a popular medical mushroom that has been widely used in oriented countries for thousands of years [18]. In a recent study, *Ganoderma* spore lipids (GSL) were extracted from sporoderm-broken germinating *Ganoderma* spores using supercritical fluid carbon dioxide. This extraction process concentrated on the major bioactive components of *G. lucidum*, including triterpenoids, polysaccharides, fatty acids, and sterols. Several studies have reported that GSL have more pharmaceutical activities than normal *G. lucidum* and show excellent anti-inflammatory and anticancer effects [19, 20]. Our previous study also demonstrated that GSL reduce cyclophosphamide-induced BMSC damage and protect against hematopoiesis [21]. However, it remains unclear whether GSL can protect BMSCs and hematopoietic cells from TPF-induced damage.

In this study, we investigated the protective effects of GSL against TPF-induced damage to BMSCs and hematopoiesis.

## Methods

### Reagents and antibody

GSL were supplied by Holistol International Ltd. (Hong Kong, China). Docetaxel, cisplatin, 5-fluorouracil, dexamethasone, ascorbic acid, β-glycerol phosphate, indomethacin, and rosiglitazone were obtained from Sigma–Aldrich (St. Louis, MO, USA). Dulbecco’s modified Eagle’s medium (DMEM), fetal bovine serum (FBS), and 0.25% trypsin/0.02% EDTA were purchased from Gibco-BRL Life Technologies (New York, NY, USA). Antibodies against CD73 (1:200, ab175396), CD90 (1:200, ab3105), CD105 (1:200, ab221675), pro-caspase-3 (1:10000, ab32499) and cleaved caspase-3 (1:1000, ab13847), β-actin (1:1000, ab8226) were purchased from Abcam (Cambridge, UK).

### Isolation and culture of BMSCs

C57BL/6 mice were euthanized by asphyxiation with CO_2_. Bone marrow cells were collected by flushing the femurs and tibias and cultured in DMEM supplemented with 10% (vol/vol) FBS and antibiotics (100 U/mL penicillin G and 100 µg/mL streptomycin). Then, the cells were seeded into normal tissue culture flasks under the condition of 37℃ and 5% CO_2_. The culture medium was changed on day 3 and non-adherent cells were carefully removed. Subsequently, the medium was replaced after 4 days and the BMSCs were used within two or three passages.

### BMSC treatment

Two passages of BMSCs were seeded in 6-well plate at a density of 3000 cells/cm^2^ and were divided into four groups: control, TPF, GSL pre-treated, and GSL co-treated. The GSL was dissolved in dimethyl sulfoxide (DMSO). The control group was treated with 1% DMSO. The TPF group was treated with docetaxel (1.5 ng/mL), cisplatin (2 µg/mL), and 5-fluorouracil (1 µg/mL) in a total volume of 80 µg. The GSL pre-treated group was treated with 0.1% GSL for 7 days followed by 2 days of 80 µg TPF at the above-defined doses. The GSL co-treated group was simultaneously treated with 0.1% GSL and 80 µg TPF at the above-defined doses for 2 days.

### Cell viability assay

Cells (1 mL) were seeded at a density of 3,000/mL in 24-well plates with 10% FBS. The medium was replaced with fresh medium. The cell number was determined by counting each sample in duplicate using a hemocytometer under an inverted microscope (Olympus, Tokyo, Japan) every 48 h for 8d.

### Flow cytometry analysis

The surface markers CD73, CD90, and CD105 in BMSC were detected using flow cytometry. BMSCs were incubated at 4℃ with primary monoclonal anti-CD73, -CD90, and -CD105 for 30 min. After washing the cells with phosphate buffered saline (PBS) supplemented with 5% fetal calf serum and 0.4% NAN_3_ (FACS buffer), they were incubated with the indicated primary antibodies for 15 min. After washing the FACS buffer, flow cytometry was performed using a flow cytometer (BD Biosciences, Franklin Lakes, NJ, USA) and analyzed using CellQuest version 5.1 (BD Biosciences). The experiments were repeated in triplicate.

### BMSC apoptosis analysis

BMSC apoptosis was analyzed using an Annexin V-FITC/propidium iodide (PI) apoptosis detection kit (BD Biosciences). BMSCs in each group were detached using trypsin/EDTA at the end of treatment. After washing with PBS, the cells were stained using the Annexin V-FITC Apoptosis Detection Kit. Apoptotic BMSCs were detected using FACS Calibur and CellQuest (BD Biosciences).

### BMSC senescence analysis

Staining of senescence-associated β-galactosidase (SA-β-GAL) was performed as previously described [22]. BMSCs in each group were fixed and then stained with β-galactosidase staining kits according to the manufacturer’s instructions (Cell Signaling Technologies, Boston, MA, USA). Images of labeled cells were captured using a microscope (Olympus) and staining intensity was quantified using ImageJ.

### ROS detection

The levels of intracellular reactive oxygen species (ROS) were measured using an ROS assay kit (Beyotime Biotechnology, S0033, Shanghai, China) according to the manufacturer’s instructions. After treatment, BMSCs in each group were incubated with serum-free medium containing 10 µM dichlorodihydrofluorescein diacetate (DCF-DA) at 37 ℃ for 30 min and washed with PBS three times. Fluorescence was observed using an inverted fluorescence microscope (Olympus). Fluorescence intensity was measured using ImageJ.

### BMSC differentiation assay

After treatment, the normal medium of each BMSC group was replaced with the differentiation medium. Osteogenic differentiation medium consisted of DMEM containing 10% FBS, 10 mM b-glycerophosphate, 1 µM dexamethasone, and 0.2 mM ascorbic acid. The medium was replaced every 3 days until day 21. Adipogenic differentiation was induced by DMEM containing 10% FBS, 2 mM L-glutamine, 1 µM dexamethasone, 500 µM 1-methyl-3-isobutylxanthine, 10 µg/mL insulin, and 100 U/mL penicillin/streptomycin; BMSCs were incubated in adipogenic induction medium for 7 days. When morphological signs of BMSC differentiation were visible, cells were fixed with polyformaldehyde for 20 min. Chondrogenic differentiation was performed using a STEMPRO Chondrogenesis Differentiation Kit (Gibco Life Technologies, Frankfurt, Germany) according to the manufacturer’s protocol. Osteogenesis, adipogenesis, and chondrogenesis were examined using Alizarin Red S, Oil Red O, and Alcian blue staining, respectively. The cells were observed under an inverted phase microscope.

### Real-time polymerase chain reaction

Total RNA from MSCs after differentiation induction was extracted using TRIzol reagent (Invitrogen, Carlsbad, CA, USA). RNA samples were converted into complementary DNA using the Superscript III First-Strand Synthesis System for Reverse Transcription-Polymerase Chain Reaction (RT-PCR) (Invitrogen) according to the manufacturer’s instructions. Gene transcripts were quantified on a 7,500 Real-time PCR System using Power SYBR Green dye (Applied Biosystems). PCR conditions were one cycle of 50 ℃ for 5 min and 95 ℃ for 10 min, followed by 50 cycles of 94 ℃ for 10 s and 60 ℃ for 1 min. The primer sequences used for RT-PCR were: glyceraldehyde-3-phosphate dehydrogenase (GAPDH) forward, 5’-tgtgatgggtgtgaaccacg-3’; GAPDH reverse, 5’-cagtgagcttcccgttcacc-3’; osteocalcin forward, 5’-tacacgtgcaggtcaatccc-3’; osteocalcin reverse, 5’-gggcagcacaggtcctaaat-3’; parathyroid hormone receptor (PTHr) forward, 5’-aagcacgaagtgggagtagc-3’; PTHr reverse, 5’-ggagccattaaggaagccgt-3’; peroxisome proliferation-activated receptor-γ (PPARγ) forward, 5’-aagcacgaagtgggagtagc-3’; PPARγ reverse, 5’-ggagccattaaggaagccgt-3’; lipoprotein lipase (LPL) forward, 5’-ccagctgggcctaactttga-3’; LPL reverse, 5’-aactcaggcagagccctttc-3’.

### Western blotting

BMSCs from each group were washed with ice-cold PBS and lysed in Laemmli sample buffer containing protease and phosphatase inhibitors. After centrifugation at 12,000 rpm for 20 min, the protein concentration of the supernatant was determined using a bicinchoninic acid assay. Equal amounts of proteins were separated by sodium dodecyl sulfate-polyacrylamide gel electrophoresis and transferred onto methanol-activated polyvinylidene difluoride membranes (Millipore, Massachusetts, USA). The membranes were blocked in Tween supplemented with 5% non-fat skim milk at room temperature for 2 h, incubated with primary antibody against caspase 3 at 4 ℃ overnight, and blotted with appropriate secondary antibody conjugated to horseradish peroxidase. Electrochemiluminescence was performed using a chemiluminescence system (Alpha, California, USA) according to the manufacturer’s instructions.

### Animal model

Forty male C57BL/6 mice were provided by the Animal Facility of Shenzhen University, Shenzhen, China. All mice were 7 weeks old and weighed 36 g. Mice were fed under standard laboratory conditions (25 ± 2 ℃, 60% ± 10% relative humidity, and 12 h light–dark cycle). All experimental procedures were approved by and conducted in accordance with the guidelines of the Institutional Animal Ethics Committee of Shenzhen University Medical School.

The mice were randomly allocated into four groups: control group (*n* = 10), TPF group (*n* = 10), GSL pre-treated group (*n* = 10), and GSL co-treated group (*n* = 10). Mice in the TPF group received TPF via intraperitoneal injection (docetaxel 20 mg/kg, cisplatin 6 mg/kg, and 5-fluorouracil 17 mg/kg). Mice in the pretreated group received 1 mg/kg GSL daily for 7 days and were intraperitoneally injected with TPF for 2 days. Mice in the co-treated group simultaneously received 1 mg/kg GSL and TPF for 2 days. The control group received equal quantities of saline by oral gavage.

### Routine blood analysis

At the end of treatment, blood samples (0.5 mL) from the posterior vena cava of CO_2_ anesthetized mice were collected into tubes containing ethylenediaminetetraacetic acid. Routine blood analysis was performed using an automatic hematology analyzer (VetAutoread, USA) for white blood cell (WBCs) and peripheral platelet (PLTs) counts.

### Hematopoietic colony-forming cell assay

Bone marrow cells were collected and cultured in Iscove’s Modified Dulbecco’s Medium supplemented with FBS, bovine serum albumin, β-mercaptoethanol, and methylcellulose. For colony-forming cell assay, the bone marrow cells (2 × 10^4^ cell per dish) were seeded into 35 mm dishes with the following growth factors: 100 ng/mL recombinant mouse SCF, 10 ng/mL recombinant mouse interleukin 3, 10 ng/mL recombinant mouse interleukin 6, 10 ng/mL mTPO, 3 U/mL recombinant human erythropoietin, 10 ng/mL recombinant human granulocyte colony-stimulating factor. After 10 days of incubation, the dishes were observed under a microscope, and the granulocyte–macrophage colony-forming units (CFU-GM), erythroid colony-forming units (CFU-E), and erythroid burst-forming units (BFU-E) were counted.

### Statistical analysis

Data are expressed as mean ± standard deviation (SD). Statistical differences were analyzed using one-way analysis of variance (ANOVA) and Tukey’s test. Statistical significance was set at *p* < 0.05. All experiments were repeated at least three times.

## Result

### GSL protects the morphology and phenotype of BMSCs from TPF-induced cytotoxicity

**Fig. 1:**
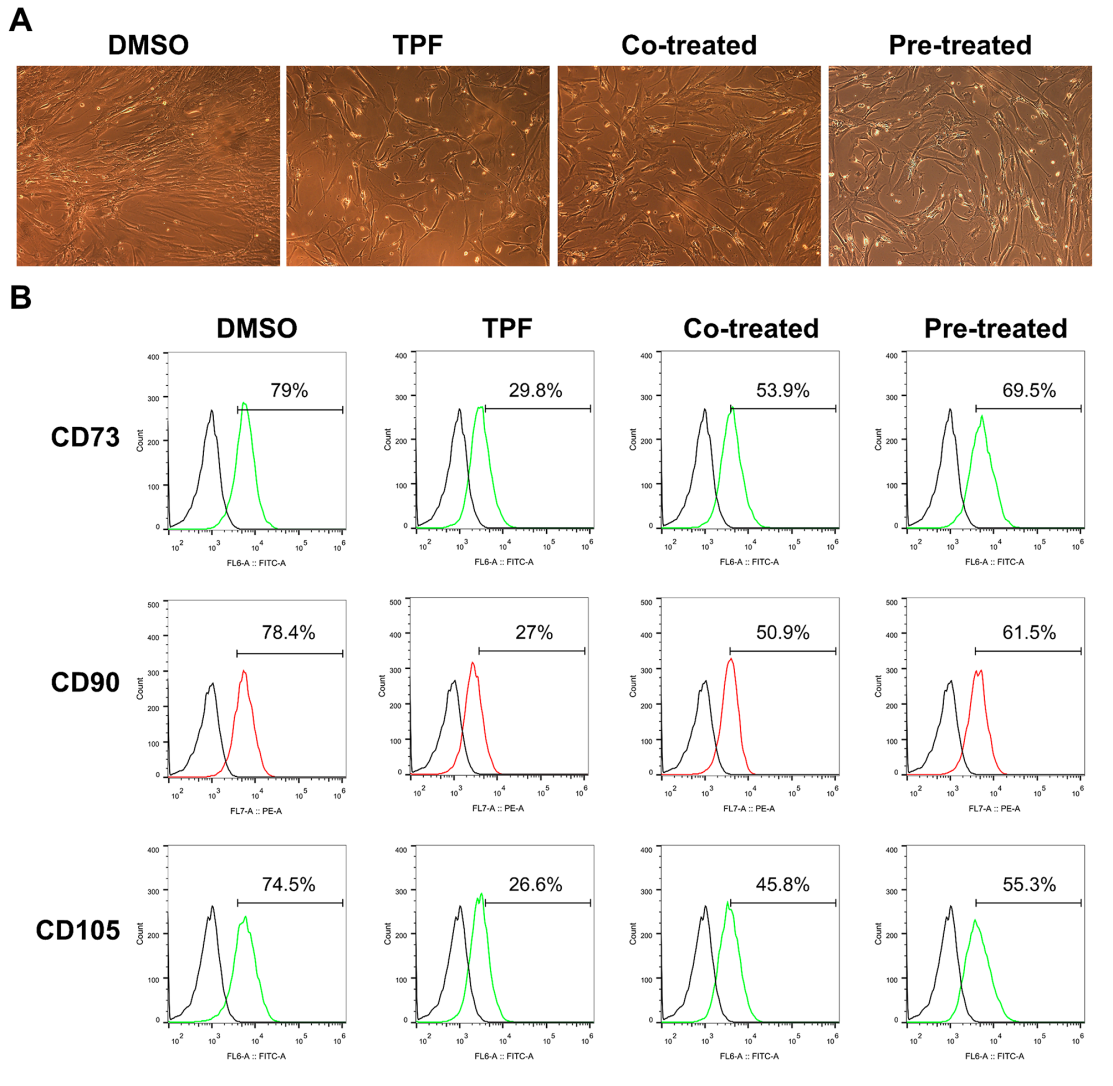
Effect of GSL on morphology and surface markers of MSCs. **A**. Representative images of morphology. The original magnification was 100×. **B**. Representative flow cytometry analysis of characteristic MSC surface markers CD73, CD90, and CD105 in the DMSO group, CTX group, Co-treated group, and Pre-treatment group

To investigate the toxicity of TPF to BMSCs and the protective effects of GSL, we first examined the morphology and phenotype of BMSCs in various groups. BMSCs in the control group displayed a typical fibroblast-like spindle morphology, whereas large proportions of BMSCs lost this typical morphology and exhibited round or irregular shapes after TPF treatment. In contrast, GSL pretreatment and co-treatment relieved TPF-induced morphological damage, with more BMSCs showing typical characteristics. Moreover, more cells in the pretreatment group had a fibroblast-like morphology than those in the co-treatment group (Fig. 1A). Flow cytometric analysis showed that the expression rates of CD73, CD90, and CD105 in the DMSO (Control) group were 79%, 78.4%, and 74.5%, respectively, whereas TPF treatment resulted in significant reductions of 29.8%, 27%, and 26.6%, respectively (Fig. 1B). In contrast, after treatment with GSL, the expression levels of these markers recovered. The expression rates of CD73, CD90, and CD105 in the co-treatment group were 53.9%, 50.9%, and 45.8%, respectively, whereas pre-treatment resulted in higher expression rates, reaching 69.5%, 61.5%, and 55.3%, respectively. These results demonstrated that GSL reduced the morphological and phenotypic changes in BMSCs induced by TPF.

### GSL relieves the damage of BMSC proliferation induced by TPF

**Fig. 2:**
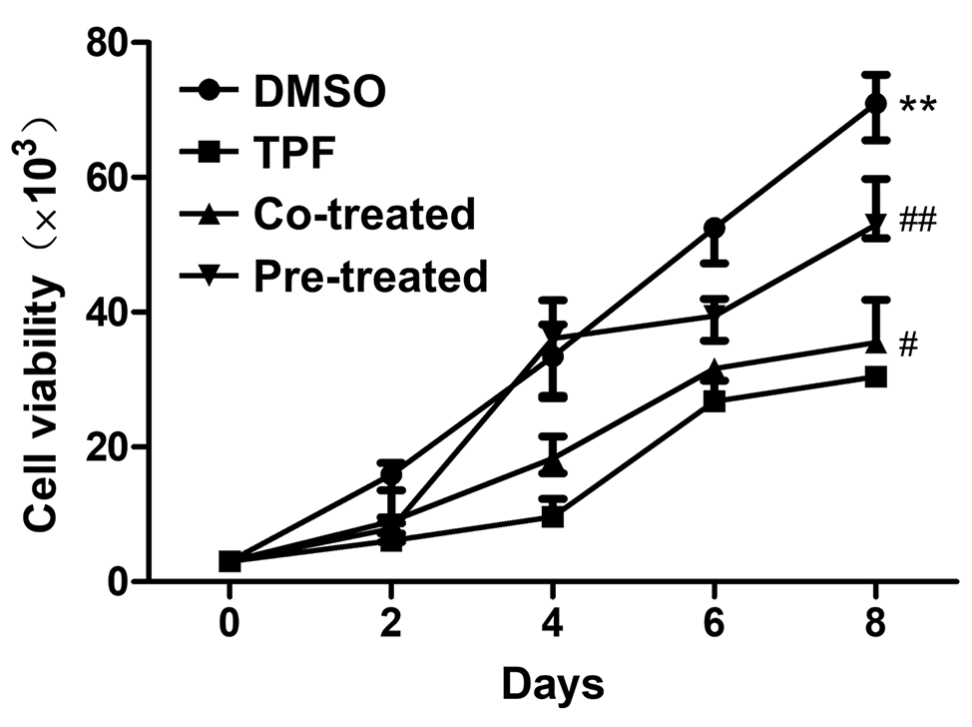
Effect of GSL on MSC proliferation. The proliferative capacity of the MSCs was measured using a cell viability assay. Data are expressed as mean ± SEM. **p < 0.01 TPF versus DMSO; #*p* < 0.05, ##*p* < 0.01, Co-treated and Pre-treated versus TPF.

To test whether GSL protect the proliferative ability of BMSCs, we used a cell viability assay to study the influence of TPF on BMSC proliferation and the protective effects of GSL. The proliferative ability of BMSCs in the TPF group was lower than that in the control group (*p* < 0.01; Fig. 2), illustrating that TPF directly impaired the viability of BMSCs. However, the TPF effect was reversed by GSL, as evidenced by a significant increase in the proliferative ability of the co-treatment and pre-treatment groups compared with the TPF group (*p* < 0.05 Co-treated vs. TPF; *p* < 0.01 Pre-treated vs. TPF). Additionally, BMSCs in the pre-treatment group showed more vigorous proliferation than those in the co-treatment group (*p* < 0.01 vs. TPF). Therefore, GSL effectively alleviated TPF-induced inhibition of BMSC proliferation.

### GSL inhibits the apoptosis of BMSCs induced by TPF

**Fig. 3:**
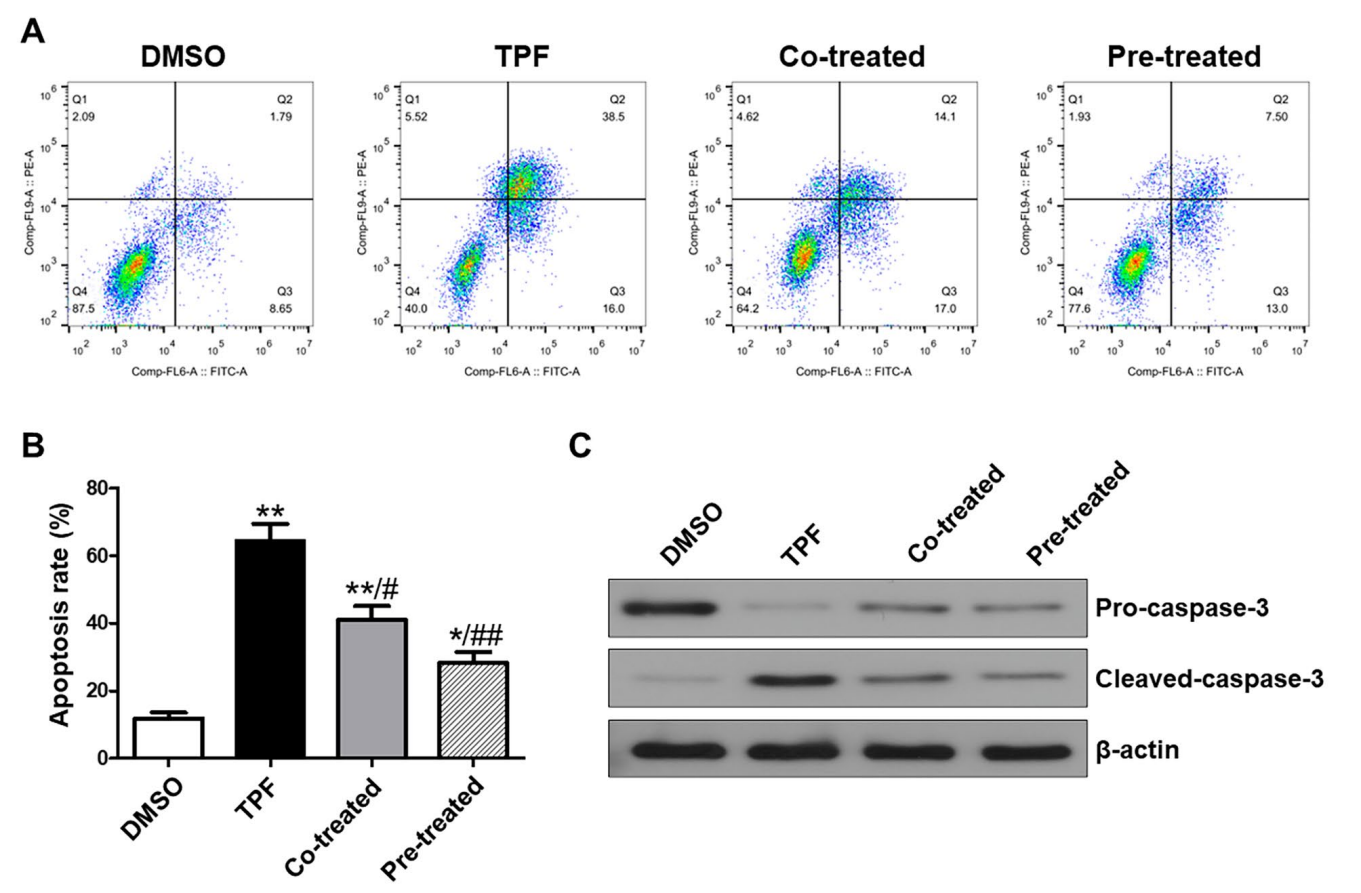
Effect of GSL on MSC apoptosis. **A, B**. Flow cytometry analysis of Annexin V/PI staining. **C**. Western blot results for pro-caspase-3 and cleaved-caspase-3 in MSCs. β-Actin served as an internal control. Data are expressed as mean ± SEM. *p < 0.05, **p < 0.01 versus DMSO; ##*p* < 0.01, Co-treated and Pre-treated versus TPF.

Annexin V/PI staining followed by flow cytometry was performed to examine TPF-induced BMSC apoptosis and the anti-apoptotic effects of GSL. The Annexin V/PI staining chart was divided into four parts. Viable cells were Annexin V (-)/PI (-), early-stage apoptotic cells were Annexin V (+)/PI (-), middle- and late-stage apoptotic cells were Annexin V (+)/PI (+), and necrotic cells and cellular debris were Annexin V (-)/PI (+) [23]. The apoptotic rate of TPF-treated BMSCs was significantly higher than that of the control group (*p* < 0.01; Fig. 3A, B). In contrast, BMSCs with pre-treatment and co-treatment showed a low apoptotic rate (*p* < 0.05 Co-treated vs. TPF; *p* < 0.01 Pre-treated vs. TPF), and GSL pre-treatment led to a lower apoptotic rate than co-treatment (*p* < 0.01 vs. TPF). To elucidate the underlying mechanisms of GSL against TPF-induced apoptosis, we measured the expression of caspase-3 in the BMSCs of all groups. Western blotting showed that TPF induced high expression of cleaved caspase-3, which was decreased by GSL pre-treatment and co-treatment (Fig. 3C). These results revealed that GSL attenuated TPF-induced BMSC apoptosis by suppressing cleaved caspase-3 expression.

### GSL inhibits premature senescence of BMSCs induced by TPF

**Fig. 4:**
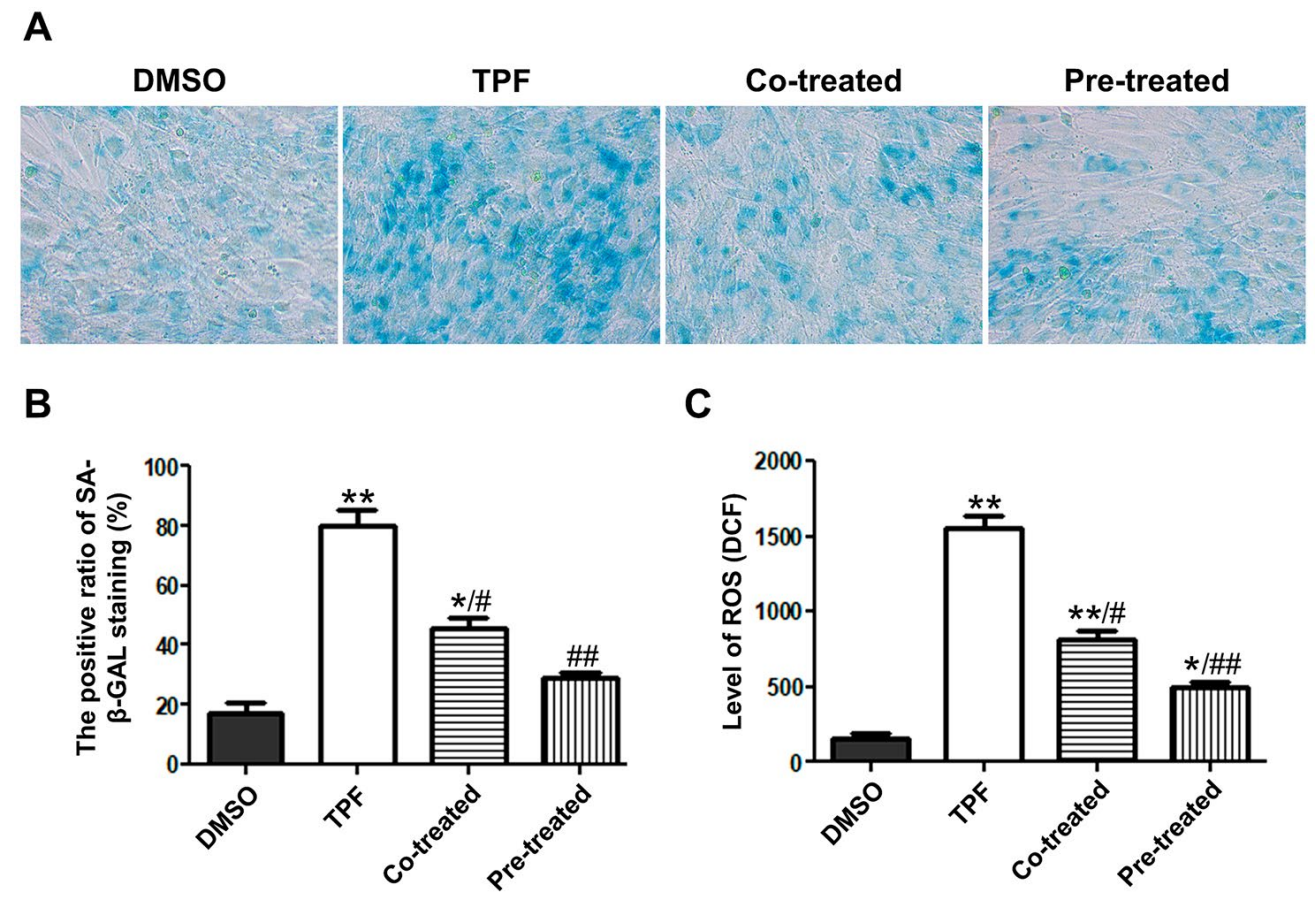
Effect of GSL on senescence and ROS of MSCs. **A**. SA-β-GAL staining to detect senescent BMSCs. Senescent cells are blue-green stained. The original magnification was 200×. **B**. Positive ratio of SA-β-GAL staining. **C**. The level of ROS measured by DCF. Data are presented as mean ± SEM. *p < 0.05, **p < 0.01 versus DMSO; #*p* < 0.05, ##*p* < 0.01, Co-treated and Pre-treated versus TPF.

Premature senescence after treatment with TPF and GSL was evaluated by staining of SA-β-GAL. We observed a significant increase of SA-β-GAL-positive cells in the TPF group (*p* < 0.01 vs. DMSO; Fig. 4A, B). In contrast, the number of SA-β-GAL-positive cells decreased in the pre-treatment and co-treatment groups (*p* < 0.05 Co-treated vs. TPF; *p* < 0.01 Pre-treated vs. TPF). Moreover, the pre-treatment group had fewer senescent cells than the co-treatment group (*p* < 0.01 vs. TPF). These results show that although TPF triggers premature senescence of BMSCs, GSL can inhibit this effect.

### GSL reduces TPF-induced ROS formation

ROS are associated with apoptosis and senescence [24]. ROS accumulation leads to DNA impairment and promotes apoptosis and senescence. The ROS content in BMSCs treated with TPF was higher than that in the DMSO group (*p* < 0.01; Fig. 4C), whereas the ROS content significantly decreased after GSL pre-treatment and co-treatment (*p* < 0.05 Co-treated vs. TPF; *p* < 0.01 Pre-treated vs. TPF). Additionally, cells pretreated with GSL showed lower ROS levels than those co-treated with GSL (*p* < 0.01 vs. TPF). These results suggested that GSL reduced TPF-induced ROS formation.

### GSL attenuates the damage of MSC differentiation induced by TPF

**Fig. 5:**
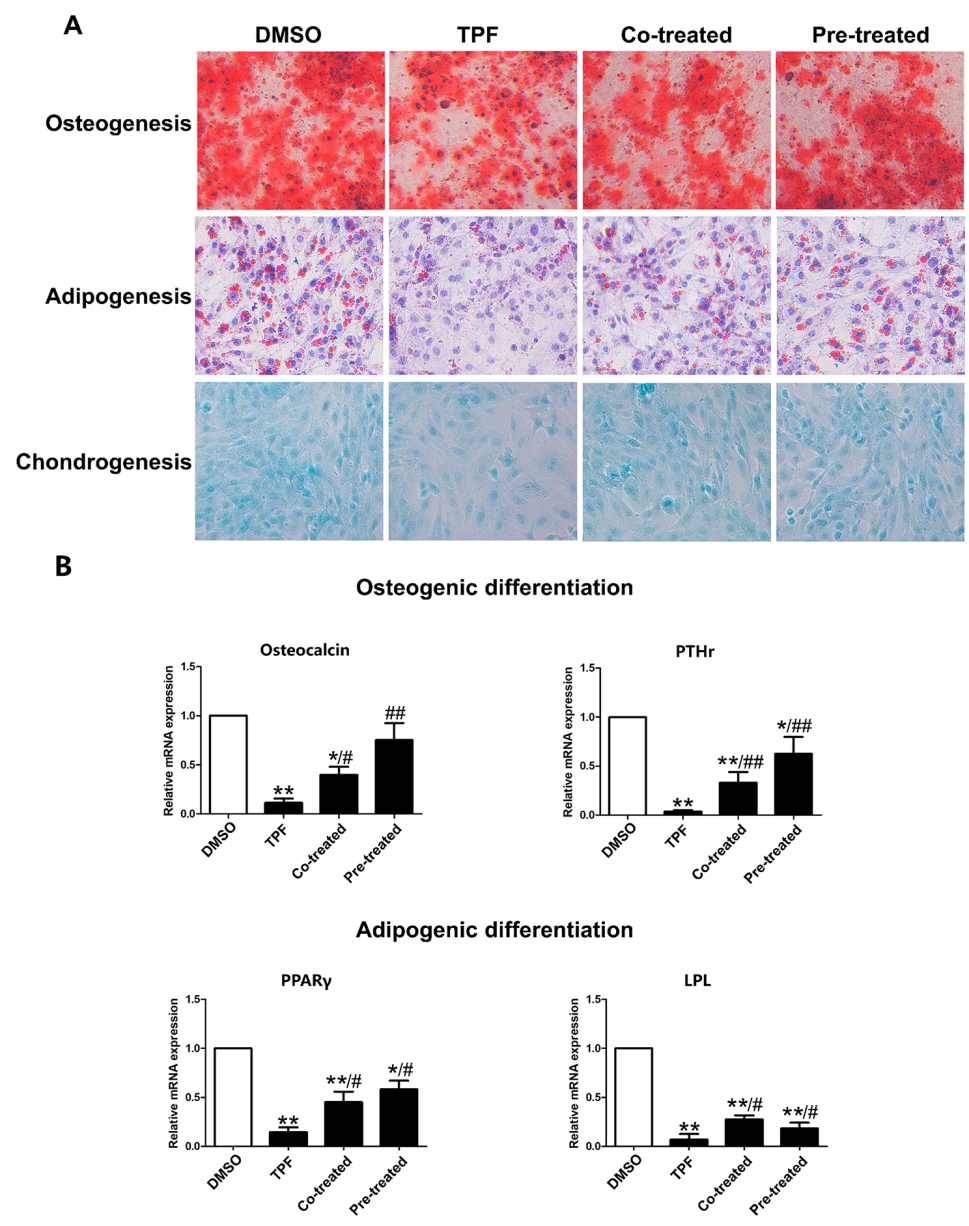
Effect of GSL on MSC differentiation potentials. **A**. Representative images of alizarin red S, oil red O, and Alcian blue staining of MSCs following osteogenic, adipogenic, and chondrogenic inductions. The original magnification was 200×. **B**. RT-PCR results of the expression of Osteocalcin, PTHr, LPL, and PPARγ. Data are expressed at mean ± SEM.*p < 0.05, **p < 0.01, versus DMSO; #*p* < 0.05, ##*p* < 0.01, Co-treated and Pre-treated versus TPF.

The ability to undergo multi-lineage differentiation is a hallmark of functional BMSCs [25]. Under specific induction conditions, BMSCs can differentiate into osteoblasts, adipocytes, and chondrocytes. After GSL and TPF treatment, BMSCs were induced to differentiate into adipocytes, osteoblasts, and chondrocytes and quantified by Alizarin Red S, Oil Red O, and Alcian blue staining. A strong reduction in induced differentiation was observed following TPF treatment. However, cotreatment and pretreatment significantly attenuated the reduction in TPF-induced osteogenic, adipogenic, and chondrogenic differentiation (Fig. 5A). Moreover, RT-PCR confirmed that TPF significantly reduced the expression of characteristic osteogenic (Osteocalcin and PTHr) and adipogenic (PPARγ and LPL) markers (*p* < 0.01 vs. DMSO; Fig. 5B), whereas GSL treatment reversed these effects (Osteocalcin, *p* < 0.05 Co-treated vs. TPF, *p* < 0.01 Pre-treated vs. TPF; PTHr, PPARγ and LPL, *p* < 0.05 vs. TPF). These results demonstrate that GSL recovered TPF-induced damage to the differentiation potential of BMSCs.

### GSL reduces the damage of hematopoiesis induced by TPF in vivo

**Fig. 6:**
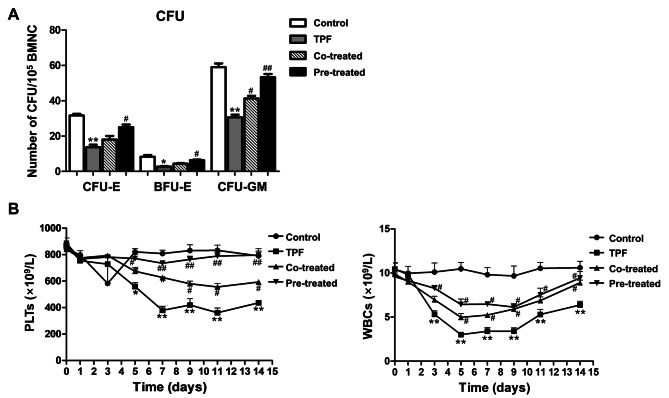
Effect of GSL on the capacity of colony-forming in mouse bone marrow cells and the peripheral blood cell counting of mice. **A**. Number of CFU-E, BFU-E, and CFU-GM. **B**. Number of PLTs and WBCs. Data are expressed at mean ± SEM.*p < 0.05, **p < 0.01 TPF versus control; #*p* < 0.05, ##*p* < 0.01, Co-treated and Pre-treated versus TPF.

Mouse models were established to estimate the protective effects of GSL on hematopoiesis in vivo. At the end of treatment, the bone marrow of mice in all groups was isolated and cultured in hematopoietic colony-forming medium to test the colony formation capacity. The number of colonies was significantly lower in the TPF group than in the control group (BFU-E, *p* < 0.05 vs. Control; CFU-E and CFU-GM, *p* < 0.01 vs. Control; Fig. 6A). However, GSL co-treatment increased the formation of CFU-GM colonies (*p* < 0.05 vs. TPF). Moreover, the pre-treated GSL group had more colonies than the co-treatment group (BFU-E, *p* < 0.05 vs. TPF; CFU-E and CFU-GM, *p* < 0.01 vs. TPF). To further investigate the protective effects of GSL in vivo, we examined peripheral PLTs and WBCs at different time points for 14 days after GSL and TPF treatment. TPF treatment led to a significant decrease in PLTs and WBCs (*p* < 0.01 vs. Control; Fig. 6B), which was reversed by GSL treatment. Pre-treatment with GSL had a better effect in recovering the PLTs number than co-treatment (*p* < 0.01 vs. TPF). These results illustrate that GSL treatment can alleviate hematopoietic damage caused by TPF.

## Discussion

Application of the TPF regimen significantly increases the effectiveness of chemotherapy in HNSCC. However, its efficacy comes at the expense of substantial toxicity, such as intestinal barrier injury and vascular endothelial dysfunction, whereas TPF-induced acute hematopoietic toxicity is particularly common [4–6, 26, 27]. Previous clinical studies have shown that most patients experience severe neutropenia after TPF treatment and require high-dose antibiotics to prevent fatal infection [3, 6, 28]. Moreover, bleeding due to thrombocytopenia is common [28]. Pancytopenia is the most severe adverse effect, which not only causes anemia, infection, and bleeding but also delays the initiation of the next cycle of chemotherapy [29]. Furthermore, long-term treatment with TPF can cause chronic bone marrow damage, which is a concern in patients with HNSCC who require multiple cycles of chemotherapy. This can result in myelosuppression as the destruction of hematopoietic stem cells progresses [30, 31]. In this study, we provide direct evidence that TPF is toxic to BMSCs and impairs hematopoiesis. These results may partially explain the hematopoietic damage observed in patients treated with TPF.

In clinical practice, Granulocyte Colony-Stimulating Factor (G-CSF) is commonly used to prevent and treat TPF-induced myelosuppression [32]. G-CSF increases the number of peripheral granulocytes and macrophages, thereby improving resistance to infection without influencing other blood cell types [32]. Additionally, G-CSF enhances the proliferation and invasion of HNSCC, potentially facilitating cancer progression [33, 34]. Therefore, there is a need to develop new drugs that reduce TPF-induced hematopoietic toxicity and myelosuppression. Several published reports have indicated that protecting BMSCs is an efficient approach for reducing chemotherapy-induced hematopoietic toxicity [12, 15, 16, 21]. In our previous study, we found that GSL effectively protect BMSCs from cyclophosphamide cytotoxicity [21]. Moreover, the bioactive components of GSL like lactones, triterpenoids, and polysaccharides, reduce chemotherapy-induced BMSCs damage and apoptosis [35]. Based on these findings, we propose that GSL may reduce the toxicity of TPF in BMSCs.

In our dataset, GSL-pretreated and co-treated BMSCs sustained a typical fibroblast-like morphology, indicating that GSL reduced the morphological damage induced by TPF. Flow cytometry showed that under the protection of GSL, the expression of the characteristic BMSC surface markers CD73, CD90, and CD105 was significantly higher than that with TPF treatment alone. CD90 and CD105 play important roles in the establishment and stability of HME. CD90 promotes the differentiation of BMSCs into osteoblasts, whereas CD105 facilitates their differentiation into chondroblasts, both of which may contribute to the establishment of HEM [36, 37]. In general, GSL protected the morphology and phenotype of BMSCs from TPF damage.

Cell proliferation is an essential indicator of cell viability. Although BMSCs have strong expansion capacity, our cell viability assay results indicated that this capacity was significantly impaired by TPF. Therefore, it is conceivable that most BMSCs are in a quiescent or inhibited state. Indeed, we observed a strong induction of apoptosis in TPF-treated BMSCs. To investigate the mechanism underlying TPF-induced apoptosis, we examined the expression of apoptosis-associated proteins. TPF-induced apoptosis was attributed to an increase in caspase-3, a key protein in the caspase pathway that promotes apoptosis [38]. However, when BMSCs were pre-treated or co-treated with GSL, we observed a higher proliferation capacity and lower apoptotic rate and caspase-3 levels than in the TPF group. These findings suggest that GSL protects against the cytotoxic effects of TPF, promotes cell proliferation, and prevents apoptosis.

In addition to TPF-induced proliferation impairment and apoptosis, we found that the degrees of SA-β-GAL staining increased in BMSCs treated with TPF, indicating the appearance of premature senescence in these cells. DNA damage is a direct factor that induces cell senescence and can be caused by various factors, such as ionizing radiation, ultraviolet light, oxidative stress, and chemical mutagens [39, 40]. Among these factors, oxidative stress plays a central role. In our dataset, TPF treatment led to an increase in the intracellular ROS content in BMSCs. ROS, including superoxide anion, hydrogen peroxide, and hydroxyl radical, contribute to oxidative stress and DNA damage, ultimately leading to cell senescence and apoptosis [39]. Therefore, TPF-induced senescence and apoptosis were strongly associated with ROS accumulation. In contrast, GSL potently delayed senescence caused by TPF by reducing ROS content. Thus, GSL possesses anti-senescence and anti-oxidation abilities that alleviate the TPF-induced impairment of BMSCs.

Adipocytes, osteoblasts, and chondrocytes, which differentiate from BMSCs, are important components of the HME and are beneficial for the regulation of HSCs [25]. Therefore, damage to the differentiation ability will undermine hematopoiesis. In the present study, we found that TPF inhibited the differentiation of BMSCs into adipocytes, osteoblasts, and chondroblasts. In contrast, GSL restored the differentiation potential of BMSCs. The number of differentiated adipocytes, osteoblasts, and chondrocytes and the expression of genes related to adipogenesis (PPARγ and LPL) and osteogenesis (Osteocalcin and PTHr) in co-treatment and pre-treatment groups were significantly higher than the BMSCs treated with TPF alone. Therefore, GSL restored the differentiation capacity of TPF-damaged BMSCs.

Animal models were established to investigate the protective effects of GSL in vivo after confirming that GSL reduced TPF toxicity in BMSCs in vitro. CFU-E and BFU-E assays show the potential of HSCs to generate erythrocytes, whereas CFU-GM has the ability to produce granulocyte–macrophage progenitors [21]. The formation assays indicated that TPF inhibited the production of erythrocytes, granulocytes, and macrophages in the bone marrow. However, this effect was reversed by GSL treatment. Hematopoiesis in the bone marrow directly influences the number of peripheral blood cells. Consistent with the colony formation results, treatment with GSL significantly restored the TPF-induced decrease in peripheral WBCs and PLTs caused by TPF treatment.

In addition, the protective effects of GSL may relate to restoring the abundance of gut microbiota. A recent study showed that *Ganoderma lucidum* polysaccharides ameliorate cisplatin and docetaxel-induced cachectic myopathy by recovering the microbiota [41]. Also, protecting gut microbiota is an important pathway to attenuate myelosuppression induced by cyclophosphamide, whereas GSL was demonstrated to remodel and restore the healthy gut microbiota [42, 43]. Therefore, further investigation on the association between TPF-induced hematopoietic damage and the recovery effect of GSL on gut microbiota is necessary.

Interestingly, the bioactive components of GSL were recently demonstrated to sensitize cancer cells to TPF [27, 44]. It has been detected that the combinational treatment of docetaxel and *Ganoderma lucidum* polysaccharide significantly increased the number of prostate cancer cells arrested in G1/S, which may associated with the inhibition of the cell cycle regulator and the dysfunction of DNA damage response pathway [45, 46]. Furthermore, the ROS/ERK pathway was identified to mediate ganoderic acid d-induced cisplatin sensibilization in ovarian tumour [27]. Overall, these studies showed the potential value of GSL in enhancing the chemotherapeutic effects of TPF. Further determining whether GSL increases the sensitivity of cancers and investigating the relevant mechanisms can effectively promote the understanding of the pharmaceutical effects of GSL.

The observed reduction in TPF-induced BMSC toxicity after GSL treatment has potential clinical implications as the TPF regimen is commonly used in HNSCC treatment. Additionally, our study and others did not find evidence of GSL toxicity in normal cells [47, 48]. GSL pretreatment was more effective than co-treatment in reducing TPF-induced BMSC damage. One possible explanation for this observation is that GSL pretreatment permits the accumulation of more anti-apoptotic and antioxidant factors before exposure to TPF, which primes the BMSCs to defend against the toxicity of TPF. Overall, this result suggests that the timing of administration is associated with the effectiveness of GSL against TPF toxicity.

## Conclusion

Collectively, our data revealed that TPF impaired BMSCs and bone marrow hematopoiesis, whereas GSL provided effective protection and reduced the damage induced by TPF. These results provide a potential adjuvant for the prevention and treatment of TPF-induced hematopoietic impairment and myelosuppression.

### Electronic supplementary material

Below is the link to the electronic supplementary material.

Supplementary Material 1: The original data for Figure 3C. The raw Western blot images of β-actin, cleavedcaspase 3 and pro-caspase 3 in groups of Control, TPF, Co-treated, and Pre-treated.

## Data Availability

All data generated and/or analyzed during this study are available from the corresponding authors upon reasonable request.

## Abbreviations

TPF: Docetaxel, Cisplatin, and 5-fluorouracil
BMSCs: Bone marrow mesenchymal stem cells
GSL: *Ganoderma* spore lipids
ROS: Reactive oxygen species
PLTs: Peripheral platelets (PLTs)
WBCs: White blood cells
HNSCC: Head and neck squamous cell carcinoma
HME: Hematopoietic microenvironment
SA-β-GAL: Senescence-associated β-galactosidase
PTHr: Parathyroid hormone receptor
PPARγ: Peroxisome proliferation-activated receptor-γ
LPL: Lipoprotein lipase
G-CSF: Granulocyte Colony-Stimulating Factor
CFU-GM: Granulocyte?macrophage colony-forming units
CFU-E: Erythroid colony-forming units
BFU-E: Erythroid burst-forming units
