# Supplementary figures and images for: Ganoderma spore lipid ameliorates docetaxel, cisplatin, and 5-fluorouracil chemotherapy-induced damage to bone marrow mesenchymal stem cells and hematopoiesis
Source: BMC Complement Med Ther. 2024 Apr 12;24:158. doi: 10.1186/s12906-024-04445-x (PMC11010295; doi:10.1186/s12906-024-04445-x)

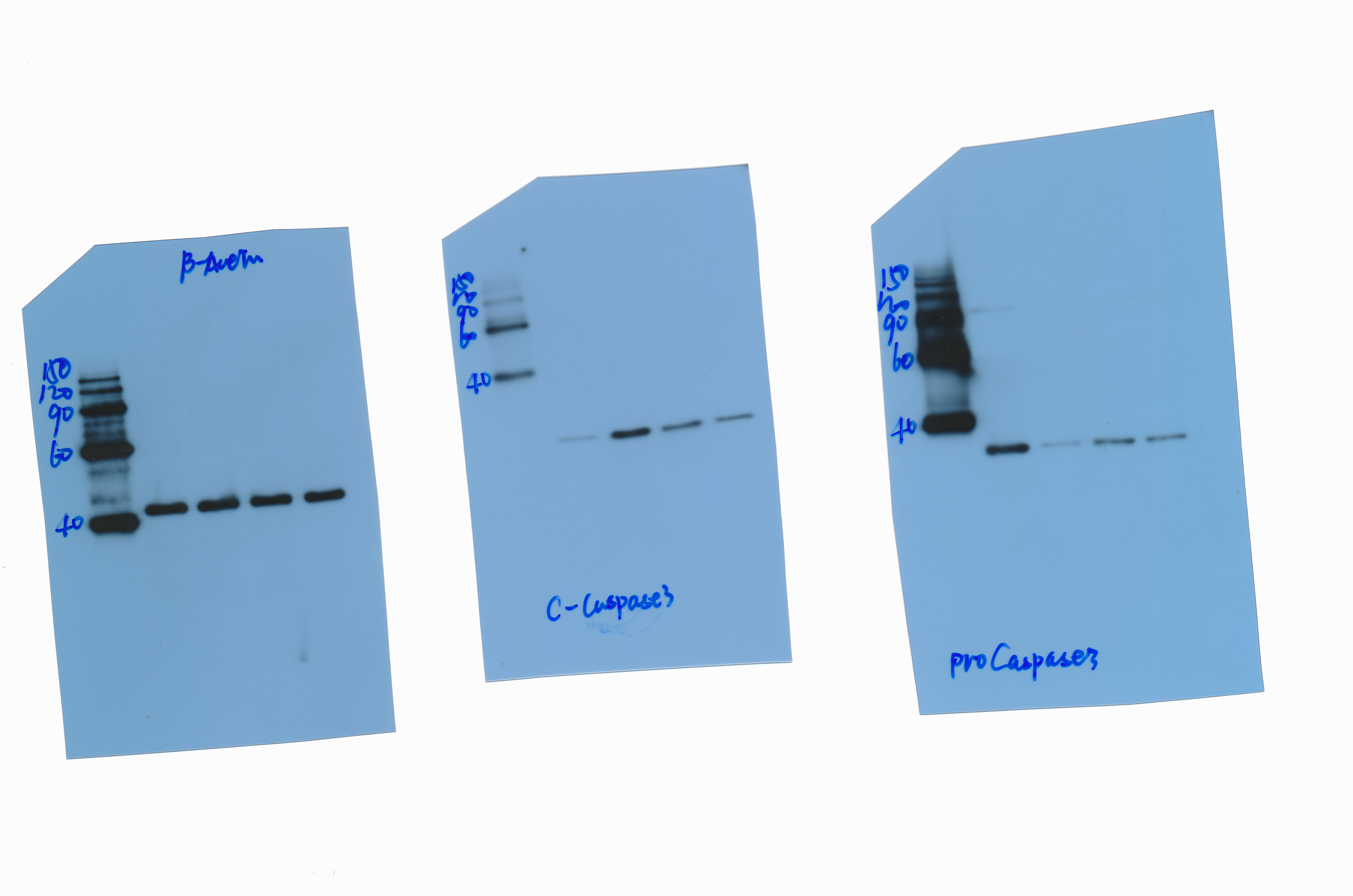

Supplement: Supplementary file 1 — Supplementary Material 1: The original data for Figure 3C. The raw Western blot images of β-actin, cleavedcaspase 3 and pro-caspase 3 in groups of Control, TPF, Co-treated, and Pre-treated. [file 12906_2024_4445_MOESM1_ESM.tif]
